# A semi-naturalistic open-label study examining the effect of prescribed medical cannabis use on simulated driving performance

**DOI:** 10.1177/02698811241229524

**Published:** 2024-02-08

**Authors:** Brooke Manning, Thomas R Arkell, Amie C Hayley, Luke A Downey

**Affiliations:** 1 Centre for Mental Health and Brain Sciences, Swinburne University of Technology, Hawthorn, VIC, Australia; 2 Institute for Breathing and Sleep (IBAS), Austin Health, Melbourne, VIC, Australia

**Keywords:** Driving performance, medical cannabis, delta-9-tetrahydrocannabinol, SDLP

## Abstract

**Background:**

Despite increasing medical cannabis use, research has yet to establish whether and to what extent products containing delta-9-tetrahydrocannabinol (THC) impact driving performance among patients. Stable doses of prescribed cannabinoid products during long-term treatment may alleviate clinical symptoms affecting cognitive and psychomotor performance.

**Aim:**

To examine the effects of open-label prescribed medical cannabis use on simulated driving performance among patients.

**Methods:**

In a semi-naturalistic laboratory study, 40 adults (55% male) aged between 23 and 80 years, consumed their own prescribed medical cannabis product. Driving performance outcomes including standard deviation of lateral position (SDLP), the standard deviation of speed (SDS), mean speed and steering variability were evaluated using the Forum8 driving simulator at baseline (pre-dosing), 2.5 h and 5 -h (post-dosing). Perceived driving effort (PDE) was self-reported after each drive. Oral fluid and whole blood samples were collected at multiple timepoints and analysed for THC via liquid chromatography-mass spectrometry.

**Results:**

A significant main effect of time was observed for mean speed (p = 0.014) and PDE (p = 0.020), with patients displaying modest stabilisation of vehicle control, increased adherence to speed limits and reductions in PDE post-dosing, relative to baseline. SDLP (p = 0.015) and PDE (p = 0.043) were elevated for those who consumed oil relative to flower-based products. Detectable THC concentrations were observed in oral fluid at 6-h post-dosing (range = 0–24 ng/mL).

**Conclusions:**

This semi-naturalistic study suggests that the consumption of medical cannabis containing THC (1.13–39.18 mg/dose) has a negligible impact on driving performance when used as prescribed.

## Introduction

The use of medical cannabis has increased significantly in recent years (Bilbao and Spanagel, 2022; Boehnke et al., 2022; Hallinan and Bonomo, 2022; Ruheel et al., 2021). Emerging, yet limited evidence suggests that delta-9-tetrahydrocannabinol (THC), the intoxicating component in cannabis that is often found in medical cannabis preparations, may have therapeutic potential for a range of refractory medical conditions, such as chronic pain, epilepsy and multiple sclerosis (Morano et al., 2020; Urtis et al., 2019). The clinical utility of THC, however, is mired by ongoing concerns around its potential to impair driving performance and cognitive function (Perkins et al., 2021). Despite patients’ prescribed cannabis consumption patterns differing from those of recreational users, research has yet to determine the relationship between THC and driving performance in patients (Schlag et al., 2021).

Although prior acute drug administration research has established that cannabis can impair driving (Hartman and Huestis, 2013), the application of these findings to patients who are receiving long-term, stable treatment with medical cannabis is uncertain (Arkell et al., 2020b). This is because previous research has primarily focused on investigating the effects of intentionally intoxicating doses of THC on healthy volunteers (see McCartney et al., 2021). Under the Australian regulatory framework, patients are given a prescription for a specific cannabinoid product, rather than the liberty to procure any form of cannabis from a dispensary. This approach ensures strict controls and medical oversight over the medicinal use of cannabis, similar to how traditional medications are prescribed, with pharmacy labels explicitly detailing dosage and frequency of use. This is in marked contrast to recreational usage, where consumption rates are typically irregular and primarily driven by the pursuit of psychoactive effects, rather than the management of health conditions and symptoms (Omare et al., 2021; Turna et al., 2020). These key differences underscore the need for research that focuses on providing safety-relevant data specifically tailored to patient populations.

Medical cannabis patients report significant enhancements across various health-related quality-of-life domains and a reduction in functional limitations over time (Arkell et al., 2023). Despite the inherent limitations due to the observational and self-reporting nature of the studies, these findings suggest that when used as prescribed for the management of underlying medical conditions, treatment with medical cannabis does not necessarily impair functional driving capabilities (MacCallum and Russo, 2018). This potential lack of adverse impact may stem from its efficacy in relieving symptoms such as pain, or improving spasticity, which may consequently reduce distractions and enhance the physical fitness required for driving (Rekand, 2014). With the effective management of chronic health conditions and potential mitigation of negative symptoms, it is plausible that changes in driving performance are limited and may even be improved in medical cannabis users (Celius and Vila, 2018).

Individuals prescribed long-term medical cannabis often report that their driving ability remains largely unaffected, leading to a reduced perception of associated risks (Wickens et al., 2019). Limited research exists on whether self-reported driving quality corresponds to actual cannabis-related changes in driving performance, with the perception of driving ability also likely to vary depending on factors such as route of administration, tolerance and frequency of use (Arkell et al., 2020a). Inhaled and oral cannabinoid products present unique pharmacokinetic profiles, which, in turn, produce variations in actual and perceived drug effects, primarily due to differences in absorption, peak and metabolic distribution (Spindle et al., 2019; Vandrey et al., 2017). Inhaled cannabinoid products have a quicker onset of action and a shorter peak effect due to the rapid absorption through the lungs, in contrast to oral products which exhibit a slower onset and longer duration of action (O’Brien and Blair, 2021). The diverse usage of medical cannabis between patient groups and varied peak effects suggest its impact on driving likely differs by product formulation and administration route (Turna et al., 2020). Further investigation is therefore needed to understand the relationship between various medical cannabis forms, their impact on driving ability and potential alterations in subjective outcomes between vaporised and oral products.

Understanding the impact of therapeutic doses of THC on driving performance is crucial for informing policy, clinical recommendations and patient education (MacCallum et al., 2021; Perkins et al., 2021). Evaluating potential risks to both the driver and other road users will contribute to preserving road safety and reducing the public health burden of traffic injuries (Tement et al., 2020). In addition, investigating the relationship between medical cannabis use and subjective changes in perceived driving ability and effort may provide further insights into self-awareness of driving ability as well as compensatory strategies employed by patients and potential tolerance effects (Sevigny, 2021).

Considering this, the present study aimed to characterise the effect of prescribed medical cannabis products on simulated driving performance, in a sample of patients with a range of chronic health conditions. Furthermore, this research explored whether medical cannabis use is associated with subjective changes in perceived driving quality or effort.

## Methods

### Participants

This semi-naturalistic study consisted of 40 adults, including 22 males (55%) and 18 females (45%), aged between 23 and 80 years (*M* = 41.38, SD ± 12.65), with an average BMI of 27.6 (SD ± 5.3). Participants were recruited via posters displayed in pharmacies and medical cannabis dispensaries throughout Melbourne, Australia. All participants had been prescribed medical cannabis containing THC for refractory conditions, including, but not limited to, sleep disorders, chronic pain, inflammatory, gastrointestinal, movement and respiratory conditions. Inclusion criteria specified that participants must be fluent in written and spoken English, possess a full driver’s license (current or expired/lapsed within the past 12 months), and be able to attend a single, in-person 7-h testing session without using medical cannabis more than once. Participants were excluded if they were pregnant, lactating or unable to abstain from illicit drug use for 7 days prior to testing. All participants provided written informed consent. The project received approval from the Swinburne University of Technology Human Research Ethics Committee and the study protocol was registered with the Australian and New Zealand Clinician Trials Registry (ACTRN12621001205820). The trial was conducted in accordance with Good Clinical Practice guidelines and the ethical standards of the Declaration of Helsinki.

### Measures

#### Driving simulator

Driving performance was evaluated using the Forum8 driving simulator at three timepoints (baseline, 2.5 h and 5 h after self-administration). Each drive comprised a 20-min highway scenario, where participants were instructed to maintain a steady lateral position in the left lane and a constant speed of 100 km/h. The simulator features an adjustable car seat, dashboard, steering wheel, indicators, brake, accelerator pedals and three integrated monitors that display a realistic scenario. The driving scenario, developed by Forum8 and customised for Australian traffic scenarios, has previously been shown to be sensitive to drug- and alcohol-related driving impairment (Aitken et al., 2023; Manning et al., 2023; Shiferaw et al., 2019). Key performance outcomes included the standard deviation of lateral position (SDLP) – a gauge of the vehicle’s lateral placement within the driving lane, often equated with the degree of vehicular weaving – in addition to standard deviation of speed (SDS), mean speed and steering variability. These outcomes are commonly employed in the evaluation of driving proficiency and are indicative of operational control and driving performance (McCartney et al., 2021; Verster and Roth, 2011).

#### Perceived driving assessment

Perceived driving quality and effort were assessed immediately following the completion of each simulated driving task. The Perceived Driving Quality Scale, developed by Verster and Roth (2012), consists of a Visual Analogue Scale (VAS) ranging from 0 = ‘I drove exceptionally poorly’ to 100 = ‘I drove exceptionally well’, with a midpoint of 50 = ‘I drove normally’. The assessment of Perceived Driving Effort (PDE) was conducted using a VAS, which ranged from 0 = ‘absolutely no effort’ to 8 = ‘extreme effort’.

### Procedure

**Figure 1.**
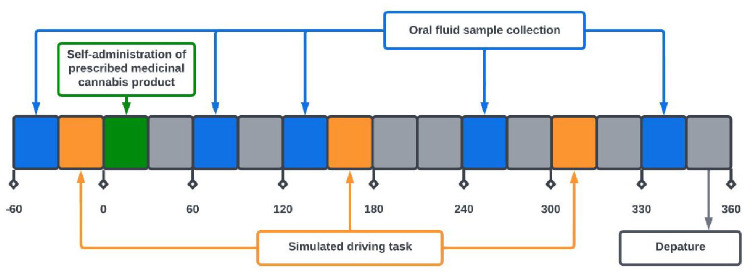
Testing day study procedure.

Participants who had consented to be contacted for further information about the study were emailed the Participant Information Consent Form and a link to an online screening questionnaire hosted on a secure web platform (Qualtrics). The first section of the questionnaire evaluated essential eligibility criteria, including age, confirmation of a current medical cannabis prescription and the ability to attend a single 7-h session with restricted cannabis use. Ineligible participants were notified, and eligible participants proceeded to the second part of the questionnaire, capturing demographic and drug use/medical history. Participants were then asked for their phone number and consent for further contact. Eligible participants were assigned a unique identifier and attended an in-person testing session at Swinburne University of Technology. Written informed consent was obtained, and demographic and medical history were confirmed by the research nurse. Participants completed baseline assessments, including biological sampling, and an online questionnaire and practiced the simulated driving task prior to self-administering their prescribed medical cannabis product under the supervision of the researchers. Drug-effect questionnaire results are presented elsewhere (Arkell et al., 2023) alongside cognitive assessments. The study schedule for the testing day is depicted in Figure 1.

In consideration of the onerous demands posed by the 7-h testing session, especially for patients with considerable health conditions, the study protocol included provisions for multiple rest periods. During these breaks, patients were offered a private space where they could recuperate and were given the option to lie down, reducing the likelihood of fatigue between tasks. Participants remained at the testing site until deemed safe to leave by the research nurse. Upon completion, participants received a handout outlining post-study restrictions, a taxi voucher to provide transport home from the testing site ($50) and monetary reimbursement for their time of $100. Participants were advised to not drive or ride a bicycle or motorbike to or from the testing site and refrain from alcohol and illicit drugs for 24 h after leaving the testing site.

### Drug administration and biological sampling

#### Cannabinoid treatments

The study protocol permitted patients to consume their medical cannabis product as prescribed on the day preceding their scheduled visit; however, they were required to abstain on the day of the testing session. Given that participants used medication for a variety of conditions, this protocol may have introduced some degree of variability with respect to the timing of the last consumption. Prior to the commencement of the testing session, the accuracy of information provided by participants regarding their prescribed medical cannabis product and dose was confirmed by two study members. This was done first by the researcher by citing a copy of each participant’s medical cannabis prescription and secondly by confirming the prescription details with the research nurse. All treatments were administered in accordance with the participant’s active prescription, although patients’ use of vaporised medical cannabis in this study may not perfectly reflect their maximum prescribed dose. The THC/ cannabidiol (CBD) dose for cannabis flower was determined using the THC/CBD percentage indicated on the product label, coupled with the mass of the plant material in milligrams. By contrast, the THC/CBD dose for oil-based products was ascertained based on the concentration in milligrams per millilitre (mg/mL) of THC/CBD in the product and the volume ingested in millilitres.

#### Oral fluid and whole blood sampling

Time-matched oral fluid and venous whole blood samples were collected at baseline, and at 1, 2, 4 and 6 h after self-administration. The Pathtech™ Oral Fluid Collection Kit was used to gather approximately 1 mL of oral fluid each time, resulting in a total of 5 mL per session. These samples were then stored at −20°C and analysed by Racing Analytical Services Limited as per the Australian/New Zealand Drug Testing Standard (AS/NZS 4308:2008). A registered research nurse obtained 10 mL of whole blood via a peripheral venous cannula, which was inserted into the participant’s non-dominant arm. Samples were immediately stored at −80°C without centrifugation and subsequently batch-shipped on dry ice to the Victorian Institute of Forensic Medicine for quantification of THC via liquid chromatography-mass spectrometry (LC-MS). Upon completion of analyses, all samples were destroyed.

### Statistical analyses

To account for variability in performance upon commencing the driving task, outcome data were analysed from when participants reached 90 km/h, as performed previously by Hayley et al. (2018). Prior to analyses, data were evaluated for completeness, with standard residuals and sensitivity analyses performed to identify and manage outliers. Linear fixed effects models with Restricted Maximum Likelihood Estimation were used to investigate differences in driving performance and perceived driving outcomes. Fixed factors included time (three levels), route of administration (two levels) and their interaction, with separate models built to investigate each outcome. The likelihood ratio statistic determined Compound Symmetry as the best-fitting covariance structure. Post hoc paired *t*-tests with planned Bonferroni adjustments for multiple comparisons were conducted to further explore differences where interaction or main effect was observed. Variables including median THC dosage split, sex and age were considered in separate models as covariates but did not account for any significant variance beyond that explained by the route of administration or product modality, and thus were not retained in the final model. Linear regressions were used to investigate associations between THC concentrations in oral fluid and whole blood with actual or perceived driving performance. All analytical procedures were two-tailed with statistical significance defined as *p* < 0.05 and were performed in SPSS (version 28).

## Results

### Participant characteristics

**Figure 2.**
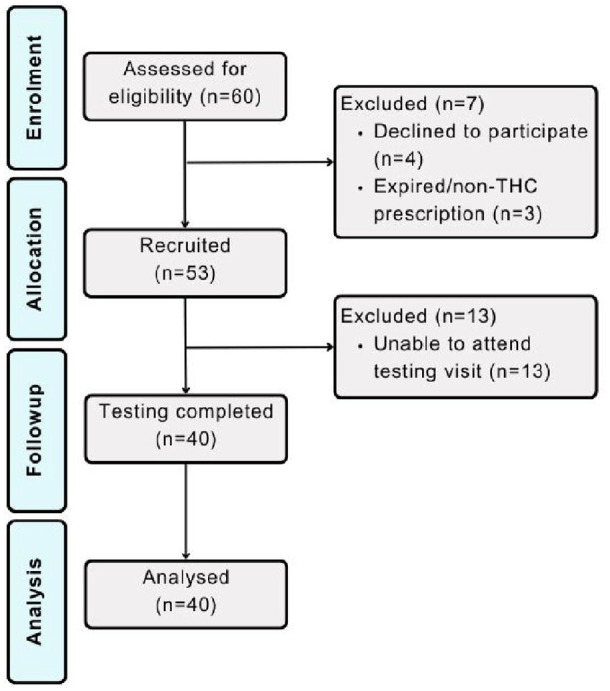
Participant CONSORT flow diagram.

Participant demographics and characteristics for the total sample (*N* = 40) are presented in Table 1, and a CONSORT diagram is depicted in Figure 2. The mean THC dose in prescribed medical cannabis products was 9.61 mg (±8.52) for oil products and 37.00 mg (±42.53) for flower products. The mean CBD dose in prescribed medical cannabis products was 9.15 mg (±10.11) for oil products and 0.38 mg (±1.58) for flower products. Most patients (92.5%) had been using their prescribed medical cannabis product for more than 30 days at their time of registration into the trial with an average treatment duration of 10.18 months (±8.73).

**Table 1.** Participant demographics and characteristics, displayed as percentage (%) or mean (standard deviation, ±SD).

Participant characteristic	*N* (%) or mean (SD)
Sex (male/female)	22/18
Age (years)	41.38 (12.66)
Body mass index (kg/m^2^)	27.60 (5.29)
Ethnicity (%)	Caucasian (95), Middle Eastern (5)
Education (%)	Secondary (25), tertiary (65), postgraduate (10)
Employment status (%)	Full-time (40), part-time/casual (25), unemployed/retired (35)
Route of administration no. (%)	Oil 23 (57.5), flower 17 (42.5)
Cannabinoid dominance no. (%)	THC dominant 23 (57.5), balanced 15 (37.5), CBD dominant 2 (5)
Indication no. (%)*	Chronic non-cancer pain 20 (50), sleep disorder 18 (45), anxiety 11 (27.5), depression 4 (10), PTSD 4 (10), gastrointestinal condition 3 (7.5), cancer pain 1 (2.5), neurological disorder 1 (2.5)
Medication no. (%)*	Antidepressants 16 (40), non-opioid analgesics 14 (35), benzodiazepines 8 (20), CNS stimulants 8 (20), opioid analgesics 5 (13), anticonvulsants 5 (13), antipsychotics 2 (5)
Alcohol consumption (%)	⩾ weekly (22.5), ⩾ monthly (47.5), < monthly (30)

THC: delta-9-tetrahydrocannabinol.

* As multiple selections could be made, cells may exceed 100%.

### Driving performance

Summary data including means and standard deviations (±) and linear fixed effects model results for simulated and perceived driving outcomes are presented in Table 2.

**Table 2.** Mean (SD) scores and linear fixed effects model results for simulated and perceived driving performance outcomes.

Outcome	Drive 1 (baseline)	Drive 2 (2.5 h)	Drive 3 (5 h)	Time	Route of administration	Time × route of administration
SDLP	29.13 (6.57)	29.60 (6.93)	28.95 (6.58)	*F*_(2,75)_ = 0.279, *p* = 0.757	***F*_(1,38)_ = 6.483,** ***p* = .015**	*F*_(2,75)_ = 0.540, *p* = 0.585
SDS	2.04 (1.07)	1.85 (0.89)	1.77 (0.73)	***F*_(2,75)_ = 3.694,** ***p* = 0.030**	*F*_(1,38)_ = 0.509, *p* = 0.480	*F*_(2,75)_ = 1.081, *p* = 0.345
Mean speed	96.88 (3.41)	96.88 (3.31)	97.70 (2.87)	***F*_(2,75)_ = 4.549,** ***p* = 0.014**	*F*_(1,38)_ = 0.408, *p* = 0.527	*F*_(2,75)_ = 1.685, *p* = 0.192
Steering variability	0.0024 (0.0016)	0.0022 (0.0015)	0.0023 (0.0019)	*F*_(2,79)_ = 0.235, *p* = 0.791	*F*_(1,37)_ = 0.990, *p* = 0.326	*F*_(2,79)_ = 1.623, *p* = 0.204
PDQ	60.59 (21.29)	55.87 (18.06)	59.00 (20.70)	*F*_(2,73)_ = 2.101, *p* = 0.130	*F*_(1,38)_ = 2.799, *p* = 0.103	*F*_(2,73)_ = 0.360, *p* = 0.699
PDE	3.64 (1.41)	3.62 (1.29)	3.03 (1.13)	***F*_(2,72)_ = 4.107,** ***p* = 0.020**	***F(1,36)* = 4.381,** ***p* = 0.043**	*F*_(2,72)_ = 1.065, *p* = 0.350

SDLP: standard deviation of lateral position; SDS: standard deviation of speed; PDQ: perceived driving quality; PDE: perceived driving effort; missing data were present for (*N* = 1) at 5 h for SDLP, SDS, mean speed and steering variability; (*N* = 1) at baseline for PDQ and PDE; (*N* = 1) at 2.5 h for PDE; and (*N* = 2) at 5 h for PDQ. Significant effects in bold.

**Figure 3.**
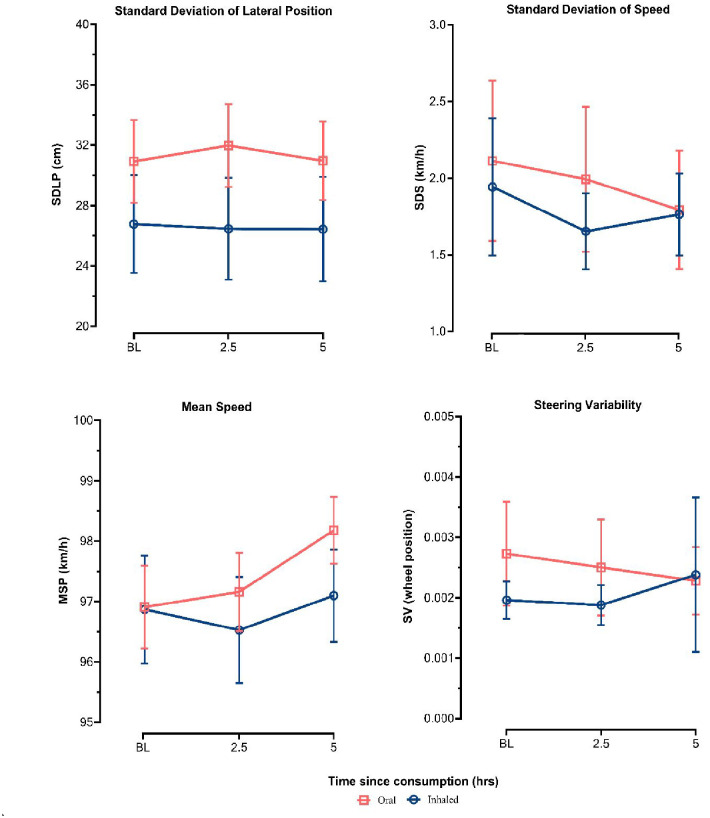
Driving performance outcomes between baseline, 2.5 h and 5 h post-administration, stratified by route of administration (error bars represent 95% confidence interval).

Differences in driving performance outcomes SDLP, SDS, mean speed and steering variability between baseline, 2.5 h and 5 h post-treatment administration are displayed in Figure 3.

A significant main effect of the route of administration on SDLP was observed (*p* = 0.015); however, the main effect of time and the time × route of administration interaction was non-significant. Post hoc comparisons revealed that patients who self-administered oil products exhibited a higher SDLP than those who used flower products at baseline (95% confidence interval [CI] 0.001–0.082, *p* < 0.05), 2.5 h (95% CI 0.015–0.096, *p* < .01) and 5 h (95% CI 0.005–0.087, *p* < 0.05).

In the case of SDS, the main effects of the route of administration and the time × route of administration interaction were non-significant. A significant main effect of time was observed for SDS (*p* = 0.030); however, post hoc comparisons over time within treatment modalities were not statistically significant after correcting for multiple comparisons.

A significant main effect of time on mean speed was observed (*p* = 0.014); however, the main effect of the route of administration and the time × route of administration interaction were non-significant. Post hoc comparisons revealed that patients’ mean speed increased at 5 h (95% CI 0.363–2.395, *p* < 0.01) and 2.5 h (95% CI 0.109–2.141, *p* < 0.05), relative to baseline. Univariate tests further revealed that mean speed predominantly increased in oil users (*p* = 0.003) rather than in patients who consumed flower products (*p* = .483).

The main effects of time, route of administration and the time × route of administration interaction were non-significant for steering variability.

### Perceived driving

**Figure 4.**
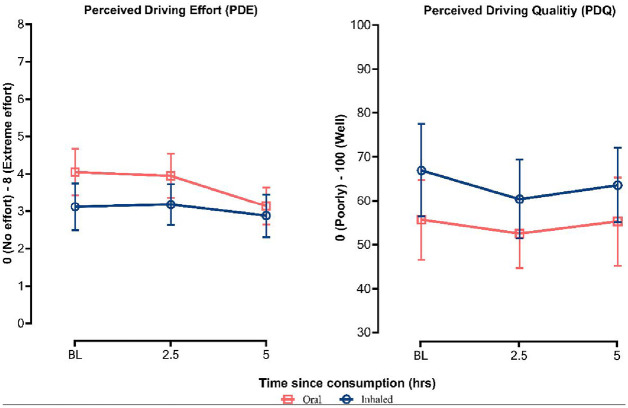
Perceived driving quality and effort between baseline, 2.5 h and 5 h post-administration, stratified by route of administration (error bars represent 95% confidence interval).

Route of administration differences in subjective PDE and quality are displayed in Figure 4.

The main effect of the time route of administration and the time × route of administration interaction were non-significant for PDQ.

In the case of PDE, a significant main effect of both time (*p* = 0.020) and route of administration (*p* = 0.043) was observed but not their interaction. Post hoc comparisons revealed that patients reported reduced PDE at 5 h relative to both baseline (95% CI −1.512 to −0.140, *p* < 0.05) and 2.5 h (95% CI −1.422 to −0.049, *p* < 0.05). Univariate tests further reveal that this reduction in PDE over time was again predominantly observed in oil users (*p* = .008) compared to patients who consumed flower products (*p* = 0.613). Post hoc comparisons also revealed that patients who consumed medical cannabis products in an oil form reported greater PDE relative to those who used flower products, predominantly at baseline (95% CI 0.122–1.734, *p* < 0.05).

### Biological sampling

**Figure 5.**
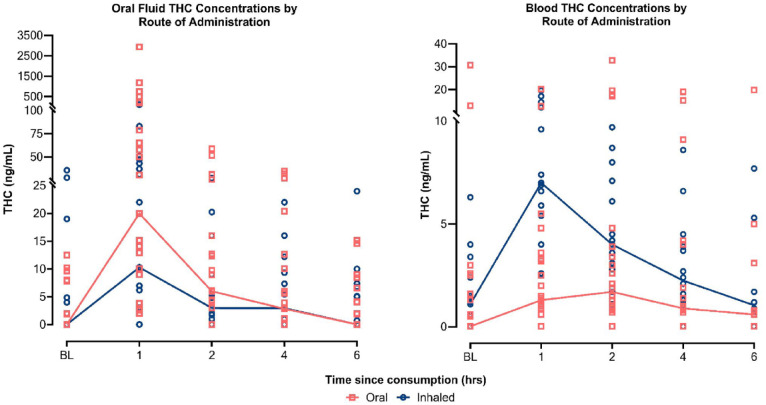
Oral fluid THC concentrations, stratified by route of administration (lines represent medians and dots represent individual patient THC concentrations showing variability across the sample). THC: delta-9-tetrahydrocannabinol.

Summary data including medians (η) and ranges for oral fluid and whole blood THC concentrations (ng/mL) are presented in Table 3. Oral fluid THC concentrations ranged from 0.00 to 2937.95 ng/mL (η = 3.0 ng/mL) across all timepoints and treatment modalities. Observable THC concentrations were present in oral fluid at baseline for 16 patients evenly split between oil and flower users (η = 7.93 vs 11.93 ng/mL, respectively). Oil products showed the greatest THC concentration variability, ranging from 0.00 to 2937.95 ng/mL at 1 h, compared to vaporised flower products which ranged from 0.00 to 101.00 ng/mL (Figure 5).

**Table 3.** Oral fluid and whole blood concentrations of THC (ng/mL) for the total sample, stratified by route of administration (oil/vaporised) and timepoint.

Timepoint	Oil				Flower				Total			
	Oral fluid		Whole blood		Oral fluid		Whole blood		Oral fluid		Whole blood	
	*N*	Median (range)	*N*	Median (range)	N	Median (range)	*N*	Median (range)	*N* *	Median (range)	*N* **	Median (range)
Baseline	22	0 (0–12.50)	22	0 (0–30.70)	17	0 (0–35.60)	15	1.10 (0–6.30)	39	0 (0–35.60)	37	0.50 (0–30.70)
1 h	23	20.00 (0–2937.95)	23	1.30 (0–20.20)	17	10.30 (0–101.00)	16	7.00 (4.00–19.60)	40	17.58 (0–2937.95)	39	3.30 (0–20.20)
2 h	23	6.00 (0–58.80)	22	1.70 (0–32.80)	17	3.00 (0–20.25)	15	4.00 (1.10–9.70)	40	4.65 (0–58.80)	37	3.00 (0–32.80)
4 h	23	2.85 (0–34.65)	19	0.90 (0–19.10)	17	2.95 (0–12.20)	14	2.25 (0–8.60)	40	2.90 (0–34.65)	33	1.40 (0–19.10)
6 h	22	0 (0–15.15)	13	0.60 (0–19.80)	16	0 (0–24.00)	8	1.05 (0–7.70)	38	0 (0–24.00)	21	0.80 (0–19.80)

THC: delta-9-tetrahydrocannabinol.

* One sample excluded at the baseline due to erroneous reporting of result (confirmed with time-matched whole blood sample); **Two missing samples occurred at 6 h (final sample) due to participant factors (needing to leave the testing site due to long commute). A total of 33 whole blood samples were not collected over a range of time points due to patient preference.

Whole blood THC concentrations ranged from 0 to 32.8 ng/mL (η = 1.5 ng/mL) across all timepoints and treatment modalities. Observable THC concentrations were present in whole blood at baseline for 21 patients across both oil (*N* = 10) and vaporised flower (*N* = 11) products (η = 2.05 vs 1.4 ng/mL, respectively). Median THC concentrations were higher for vaporised products overall and across all time points (3.05 ng/mL) relative to oil products (0.9 ng/mL). Oil products, however, showed greater THC concentration variability, ranging from 0.00 to 32.8 ng/mL with the greatest variation at 2 h, compared to flower products which ranged from 0.00 to 19.6 ng/mL with the greatest variation at 1 h.

## Discussion

In this open-label semi-naturalistic study, simulated and perceived driving performance among 40 patients was assessed prior to and following self-administration of their own prescribed medical cannabis product. While oil users tended to have higher SDLP values, this was stable over time and there was no evidence of impairment for either administration route. Furthermore, the lack of changes in speed variability suggests a modest but sustained stabilisation of vehicle control. Interestingly, there was also a significant increase in average speed at 5 h, in comparison to both baseline and the 2.5 h mark, with this increase in speed reflecting a greater accuracy in adhering to the assigned speed limit. Critically, no notable evidence of driving *impairment* (i.e. a significant decline in driving performance metrics within the simulated driving scenario) was observed for either consumption modality, relative to baseline.

Contrary to our findings, Di Ciano et al. (2020) observed reductions in mean speed during simulated driving scenarios requiring higher cognitive load but not during highway driving conditions. Preceding studies primarily involving cannabis naïve users have also more frequently associated acute cannabis use with an increase in speed variability and a general decline in average speed when using smoked or vaporised forms of cannabis (Brands et al., 2019; Lenné et al., 2010; Ronen et al., 2008). Decreases in average speed are often inversely and dose dependently related to THC concentration. A comprehensive review by Neavyn et al. (2014) suggests that this behaviour is largely attributed to drivers’ attempts to compensate for attention lapses when they are conscious of the subjective effects linked with cannabis-induced intoxication. In our study, a marginal increase in average speed was observed, this increase did not exceed the instructed speed limit of 100 km/h. This increment of <1 km/h could also be more simply attributed to participants’ desire to complete the task swiftly after a long day, despite the scenario being timed rather than distance based. This contrasts with the habitual over-cautious driving behaviour of cannabis-intoxicated individuals, who typically exhibit compensatory or unnecessary reductions in speed. Notably, such caution is more characteristic of occasional cannabis users who are acutely intoxicated. By contrast, patients in our study exhibited only mild intoxication, potentially reflecting increased tolerance or familiarity with cannabis’ effects (Ramaekers et al., 2020). This may explain the relatively stable speed and reduced likelihood of speed variability exhibited, which aligns with findings by Doroudgar et al. (2018) and suggests an adaptation to driving demands and a departure from the heightened caution seen in less frequent users.

The absence of a discernible increase in SDLP, a measure frequently considered sensitive in assessing driving performance (Hartman and Huestis, 2013), was somewhat unexpected. This is especially so, given substantial prior research demonstrating that THC consumption reliably increases SDLP in both simulator and on-road paradigms (Arkell et al., 2020b; Bosker et al., 2012; Hartman et al., 2015; Ramaekers et al., 2000). Even at relatively low THC dosages of 5 mg, a statistically significant, although not ecologically substantial, increase in vehicle weaving remains discernible compared to placebo (Manning et al., 2023). These dosages are markedly below those used in the current study (9.61 mg for oil products vs 37.00 mg for flower products), and also significantly lower than those commonly employed in a myriad of other experimental driving studies (Arkell et al., 2020b).

To contextualise our findings within an ecological framework, it is noted that an increase in SDLP of 2.4 cm is generally considered ‘ecologically significant’, as this degree of deviation is comparable to that observed at a blood alcohol concentration of 0.05% (Verster and Roth, 2011). Noteworthily, absolute SDLP values in this patient group across all drives (spanning 28.95–29.13 cm) display little difference compared to what has been recorded in healthy volunteers during a placebo condition (27.41 cm) under identical driving scenario conditions and similar demographic characteristics (Manning et al., 2023). Differences in SDLP between healthy placebo-controlled volunteers and the medical cannabis patients in our study, along with the fluctuations in SDLP among these patients over time, did not meet this established ‘ecologically significant’ threshold.

Interestingly, patients who self-administered oil products exhibited a higher SDLP overall relative to those who used flower products. The persistence of differences in SDLP at baseline suggests that the variations observed between treatment modalities may not be directly attributable to product exposure. Initially, those using oil-based products reported exerting significantly more effort while driving relative to those using flower-based products. This difference gradually declined, with oil users reporting a considerable decrease in effort by their final drive, aligning them more closely with flower product users. This change indicates that for those consuming oil products, the perceived mental or physical demand for driving decreased over time. This reduction in perceived effort during later drives could be attributed to adaptation to the oil-based product’s effects, or simply greater familiarity with the driving task; although it is also possible that flower users may have already become accustomed to the effects of their prescribed vaporised product prior to the post-dosing driving tasks. It is important to consider that factors such as individual differences in drug metabolism and tolerance may influence self-reported perceived effort levels and that these self-reported effort levels do not necessarily correlate with actual driving performance.

The prolonged acute intoxication associated with oral THC products and the more gradual onset of effects may partly explain the differences in drug-effect profiles (Vandrey et al., 2017). Users who consumed their treatment via vaporisation may have experienced diminished drug effects by the time they commenced the driving task. Variations in daily or nightly usage patterns could also have impacted these results, as patients who ingest their medical cannabis orally the night before (i.e. as a sleep aid) may have experienced residual effects the following morning. The observed disparities in baseline performance between different routes of administration may also be partially attributed to the high variability in both oral fluid and whole blood THC concentrations, particularly among oil users.

A previous report indicated that driving impairment following THC vaporisation may linger for up to 4.5 h in healthy users (Marcotte et al., 2022). By contrast, the utilisation of medical cannabis for intractable health conditions may attenuate the adverse impacts on cognitive and psychomotor functions often associated with the clinical symptoms of these conditions (Makhoul and Jankovic, 2023). This is particularly evident in chronic cannabis users who exhibit fewer neurocognitive deficits compared to infrequent users (Ramaekers et al., 2009). Existing research supports these findings, albeit derived primarily from self-reported evidence, which suggests a therapeutic role for medical cannabis in managing clinical symptoms commonly associated with cognitive and psychomotor impairment (Eadie et al., 2021; MacCallum et al., 2022). Specifically, patients who routinely administer medical cannabis for chronic conditions may develop a tolerance to the prototypical THC-induced impairments in driving abilities that are more pronounced in occasional or inexperienced users, thereby potentially reducing the impact of THC on driving competency seen in otherwise healthy subjects (Celius and Vila, 2018; Freidel et al., 2015; Veldhuijzen et al., 2006).

Prior research suggests that recreational and/or infrequent THC use can impair driving performance; however, this effect is thought to be less pronounced in habitual users, particularly patients who adhere to a stable and regular dose of a prescribed medical cannabis product (MacCallum and Russo, 2018). It is posited that tolerance to the effects of THC among chronic users may result in significantly reduced intoxication and/or impairment with a given dose of THC relative to occasional cannabis users (Doroudgar et al. 2018; Ramaekers et al., 2021). A similar mitigation of impairment could therefore extend to medical cannabis patients who are undergoing long-term treatment and adhere to maintaining a steady dosing regimen. As with other psychoactive medications, consistent and stable dosing may minimise instances of impaired driving, with tolerance development further mitigating potential impacts on driving competence (Verster and Mets, 2009). The effective management of chronic health conditions may offset potential negative symptomatic effects, leading to limited changes or even improvements in driving performance (Celius and Vila, 2018).

Outside of treatment effects related to dose, mode and timing of acute cannabis administration, the development of cannabis tolerance may attenuate certain acute driving and psychomotor performance deficits (Sevigny, 2021). More recent evidence from higher dose and more frequent cannabis users suggests the role of pharmacodynamic models of tolerance, where repeated cannabis exposure leads to neuroadaptive responses dulling acute effects (Colizzi and Bhattacharyya, 2018; Ramaekers et al., 2020). The absence of clear driving impairment in the current study, despite performance variations between routes of administration, likely indicates at least some mitigation of drug effects due to symptom relief and a potential acquired tolerance to THC’s impairing effects. It remains important to consider that the observed effects may be, in part, influenced by patients being highly motivated to demonstrate their driving is not impaired by cannabis. While this suggests that patients can overcome THC-related impairment that might have otherwise been present in an observational study setting, this still may not fully represent the real-world driving scenario, where such motivation to demonstrate unimpaired driving is less pronounced. Lastly, an absence of observable driving impairment could be due to the relatively low THC doses in medical cannabis products consumed or that patients’ driving abilities were compromised prior to consumption of their medical cannabis product.

### Limitations and future research

This study is not without its methodological limitations, which warrant consideration. Firstly, the semi-naturalistic, open-label design, whilst ecologically valid, introduces high variability in prescribed medical cannabis products consumed, patterns of prescribed use, indications for use and use of other prescribed medication. It is important to highlight the high level of concomitant medication use in this patient sample, especially medications that can themselves impair driving, such as antidepressants, benzodiazepines and opioids (Aitken et al., 2023; Dassanayake et al., 2012). We acknowledge that the use of these medications in combination with medical cannabis may produce additive or synergistic effects, although one would expect particularly pronounced driving impairment in the case of any such interactive effects. By including participants who were using other potentially impairing medications alongside medical cannabis, we hoped to maximise generalisability to the broader medical cannabis-using population and generate data with real-world relevance.

Secondly, the absence of a placebo control group indeed limits our ability to discriminate between baseline performance and the potential residual effects of medical cannabis; however, it would be neither conceptually sound nor ethically appropriate to deprive these patients of using their medication as therapeutically indicated. The absence of a testing interval shortly after cannabis vaporisation is another limitation, as peak impairment typically occurs within the first 60 min following cannabis inhalation (Arkell et al., 2019). Consequently, we acknowledge that impairment may have already subsided by this point. Lastly, we acknowledge that the low complexity and task-level monotony of the driving scenario may have concealed the full magnitude of effects on lateral vehicle control and does not reflect the full spectrum of driving challenges. Nevertheless, this approach replicates the on-road driving paradigm utilised by Ramaekers (2017, which allows for a standardised analysis of THC’s influence on fundamental driving abilities. Information regarding where and how medical cannabis users get into collisions is non-existent as epidemiological data cannot discriminate medicinal from recreational THC. While the driving scenario in our study may have been less complex, it was intentionally chosen for its high reliability in identifying driving impairment due to drug effects (Verster and Roth, 2017).

Despite these limitations, this research provides a foundation for future investigations into the effects of medical cannabis on driving performance and offers preliminary evidence suggesting minimal to no impairing effects on objective driving performance when medical cannabis is used as prescribed. Future research should endeavour to further clarify the nuanced effects of medical cannabis use on driving behaviour, particularly regarding the impact of therapeutically relevant THC doses on measures of driving performance and associated psychomotor control in clinical populations. It is essential, however, that these studies consider the effects from the onset of use over a more extended period (McCartney et al., 2021). Future investigations would also greatly benefit from integrating more complex driving tasks to detect potential medical cannabis effects with enhanced sensitivity.

## Conclusion

Overall, this semi-naturalistic study suggests that medical cannabis, used as prescribed, has a negligible impact on simulated driving performance. Despite the absence of observable driving impairment within the present scenario, patients had detectable concentrations of THC in their oral fluid for a duration of up to 6 h. By focusing on patients consuming prescribed THC-containing products at therapeutic doses, this study provides critical safety and clinically relevant data that is more representative of real-world medical cannabis use and its potential impact on driving performance. Larger and more controlled trials are necessary to validate and confirm these findings in establishing more definitive conclusions regarding road safety.

## References

[bibr1-02698811241229524] Aitken B Hayley AC Ford TC , et al. (2023) Driving impairment and altered ocular activity under the effects of alprazolam and alcohol: A randomized, double blind, placebo-controlled study. Drug Alcohol Depend 251: 110919. DOI: 10.1016/j.drugalcdep.2023.110919. 37611483

[bibr2-02698811241229524] Arkell TR Downey LA Hayley AC , et al. (2023) Assessment of medical cannabis and health-related quality of life. JAMA Netw Open 6: e2312522. DOI: 10.1001/jamanetworkopen.2023.12522. PMC10170337 37159196

[bibr3-02698811241229524] Arkell TR Lintzeris N Kevin RC , et al. (2019) Cannabidiol (CBD) content in vaporized cannabis does not prevent tetrahydrocannabinol (THC)-induced impairment of driving and cognition. Psychopharmacology 236: 2713–2724. DOI: 10.1007/s00213-019-05246-8. 31044290 PMC6695367

[bibr4-02698811241229524] Arkell TR Lintzeris N Mills L , et al. (2020a) Driving-related behaviours, attitudes, and perceptions among Australian medical cannabis users: results from the CAMS 18-19 survey. Accid Anal Prev 148: 105784. DOI: 10.1016/j.aap.2020.105784. 33017729

[bibr5-02698811241229524] Arkell TR Vinckenbosch F Kevin RC , et al. (2020b) Effect of cannabidiol and Δ9-tetrahydrocannabinol on driving performance: A randomised clinical trial. JAMA 324: 2177–2186. DOI: 10.1001/jama.2020.21218. 33258890 PMC7709000

[bibr6-02698811241229524] Bilbao A Spanagel R (2022) Medical cannabinoids: A pharmacology-based systematic review and meta-analysis for all relevant medical indications. BMC Med 20: 259. DOI: 10.1186/s12916-022-02459-1. 35982439 PMC9389720

[bibr7-02698811241229524] Boehnke KF Dean O Haffajee RL , et al. (2022) U.S. trends in registration for medical cannabis and reasons for use from 2016 to 2020: An observational study. Ann Internal Med 175: 945–951. DOI: 10.7326/M22-0217. 35696691 PMC10233658

[bibr8-02698811241229524] Bosker WM Theunissen EL Conen S , et al. (2012) A placebo-controlled study to assess standardized field sobriety tests performance during alcohol and cannabis intoxication in heavy cannabis users and accuracy of point of collection testing devices for detecting THC in oral fluid. Psychopharmacology 223: 439–446. DOI: 10.1007/s00213-012-2732-y. 22581391 PMC3456923

[bibr9-02698811241229524] Brands B Mann RE Wickens CM , et al. (2019) Acute and residual effects of smoked cannabis: Impact on driving speed and lateral control, heart rate, and self-reported drug effects. Drug and Alcohol Dependence 205: 107641. DOI: 10.1016/j.drugalcdep.2019.107641. 31678833

[bibr10-02698811241229524] Celius EG Vila C (2018) The influence of THC: CBD oromucosal spray on driving ability in patients with multiple sclerosis-related spasticity. Brain Behav 8: e00962. DOI: 10.1002/brb3.962. PMC5943754 29761015

[bibr11-02698811241229524] Colizzi M Bhattachayya S (2018) Cannabis use and the development of tolerance: A systematic review of human evidence. Neurosc Biobehav Rev 93: 1–25. DOI: 10.1016/j.neubiorev.2018.07.014. 30056176

[bibr12-02698811241229524] Dassanayake T Michie P Carter G , et al. (2012) Effects of benzodiazepines, antidepressants, and opioids on driving. Drug Safety 34: 125–156. DOI: 10.2165/11539050-000000000-00000. 21247221

[bibr13-02698811241229524] Di Ciano P Matamoros A Matheson J , et al. (2020) Effects of therapeutic cannabis on simulated driving: A pilot study. Journal of Concurrent Disorders 2: 3–13. DOI: 10.54127/DKWR5604.

[bibr14-02698811241229524] Doroudgar S Chuang HM Bohnert K , et al. (2018) Effects of chronic marijuana use on driving performance. Traffic Injury Prev 19: 680–686. DOI: 10.1080/15389588.2018.1501800. 30411981

[bibr15-02698811241229524] Eadie L Lo LA Christiansen A , et al. (2021) Duration of neurocognitive impairment with medical cannabis use: A scoping review. Front Psychiatry 12: 638962. DOI: 10.3389/fpsyt.2021.638962. 33790818 PMC8006301

[bibr16-02698811241229524] Freidel M Tiel-Wilck K Schreiber H , et al. (2015) Drug-resistant MS spasticity treatment with Sativex® add-on and driving ability. Acta Neurol Scand 131: 9–16. DOI: 10.1111/ane.12287. 25208898

[bibr17-02698811241229524] Hallinan CM Bonomo YA (2022) The rise and rise of medicinal cannabis, what now? Medicinal cannabis prescribing in Australia 2017-2022. Int J Environm Res Public Health 19: 9853. DOI: 10.3390/ijerph19169853. PMC9408026 36011488

[bibr18-02698811241229524] Hartman RL Brown TL Milavetz G Spurgin A , et al. (2015) Cannabis effects on driving lateral control with and without alcohol. Drug Alcohol Depend 154: 25–37. DOI: 10.1016/j.drugalcdep.2015.06.015. 26144593 PMC4536116

[bibr19-02698811241229524] Hartman RL Huestis MA (2013) Cannabis effects of driving skills. Clin Chem 59: 478–492. DOI: 10.1373/clinchem.2012.194381. 23220273 PMC3836260

[bibr20-02698811241229524] Hayley AC Green M Downey LA , et al. (2018) The acute and residual effects of escalating, analgesic-range doses of ketamine on driving performance: A simulator study. Prog Neuropsychopharmacol Biol Psychiatry 86: 83–88. DOI: 10.1016/j.pnpbp.2018.05.015. 29782960

[bibr21-02698811241229524] Lenné MG Dietze PM Triggs TJ , et al. (2010) The effects of cannabis and alcohol on simulated arterial driving: Influences of driving experience and task demand. Accid Anal Prev 42: 859–866. DOI: 10.1016/j.aap.2009.04.021. 20380913

[bibr22-02698811241229524] MacCallum CA Lo LA Boivin M (2021) ‘Is medical cannabis safe for my patients?’ A practical review of cannabis safety considerations. Eur J Intern Med 89: 10–18. DOI: 10.1016/j.ejim.2021.05.002. 34083092

[bibr23-02698811241229524] MacCallum CA Lo LA Pistawka CA , et al. (2022) A clinical framework for assessing cannabis-related impairment risk. Front Psychiatry 13: 883517. DOI: 10.3389/fpsyt.2022.883517. 35832600 PMC9272752

[bibr24-02698811241229524] MacCallum CA Russo EB (2018) Practical considerations in medical cannabis administration and dosing. Europ J Intern Med 49: 12–19. DOI: 10.1016/j.ejim.2018.01.004. 29307505

[bibr25-02698811241229524] Makhoul K Jankovic J (2023) Driving impairment in movement disorders. Mov Disord Clin Pract 10: 369–381. DOI: 10.1002/mdc3.13676. 36949799 PMC10026316

[bibr26-02698811241229524] Manning B Hayley AC Catchlove S , et al. (2023) Effect of CannEpil on simulated driving performance and co-monitoring of ocular activity: a randomised controlled trial. J Psychopharmacol 37: 472–483. DOI: 10.1177/02698811231170360. 37129083 PMC10184186

[bibr27-02698811241229524] Marcotte TD Umlauf A Grelotti DJ , et al. (2022) Driving performance and cannabis users’ perception of safety: A randomized clinical trial. JAMA Psychiatry 79: 201–209. DOI: 10.1001/jamapsychiatry.2021.4037. 35080588 PMC8792796

[bibr28-02698811241229524] McCartney D Arkell TR Irwin C , et al. (2021) Determining the magnitude and duration of acute Δ9-tetrahydrocannabinol (Δ9-THC)-induced driving and cognitive impairment: A systematic and meta-analytic review. Neurosci Biobehav Rev 126: 175–193. DOI: 10.1016/j.neubiorev.2021.02.003. 33497784

[bibr29-02698811241229524] Morano A Fanella M Albini M , et al. (2020) Cannabinoids in the treatment of epilepsy: Current status and future prospects Neuropsychiatr Dis Treat 16: 381–396. DOI: 10.2147/NDT.S203782. 32103958 PMC7012327

[bibr30-02698811241229524] Neavyn MJ Blohm E Babu KM , et al. (2014) Medical marijuana and driving: A review. J Med Toxicol 10: 269–279. DOI: 10.1007/s13181-014-0393-4. 24648180 PMC4141931

[bibr31-02698811241229524] O’Brien K Blair P (2021) Routes of administration, pharmacokinetics, and safety of medicinal cannabis. In: Medical cannabis and CBD in mental healthcare. Cham: Springer. DOI: 10.1007/978-3-030-78559-8_11.

[bibr32-02698811241229524] Omare MO Kibet JK Cherutoi JK , et al. (2021) Current trends in the use of cannabis sativa: Beyond recreational and medicinal application. Open Access Lib J 8: 1–15. DOI: 10.4236/oalib.1107132.

[bibr33-02698811241229524] Perkins D Brophy H McGregor IS , et al. (2021) Medical cannabis and driving: The intersection of health and road safety policy. Int J Drug Policy 97: 103307. DOI: 10.1016/j.drugpo.2021.103307. 34107448

[bibr34-02698811241229524] Ramaekers J Kauert G Theunissen E , et al. (2009) Neurocognitive performance during acute THC intoxication in heavy and occasional cannabis users. J Psychopharmacol 23: 266–277. DOI: 10.1177/0269881108092393. 18719045

[bibr35-02698811241229524] Ramaekers JG Mason NL Theunissen EL (2020) Blunted highs: Pharmacodynamic and behavioral models of cannabis tolerance. Europ Neuropsychopharmacol 36: 191–205. DOI: 10.1016/j.euroneuro.2020.01.006. 32014378

[bibr36-02698811241229524] Ramaekers JG Mason NL Kloft L , et al. (2021) The why behind the high: Determinants of neurocognition during acute cannabis exposure. Nat Rev Neurosci 22(7): 439–454. DOI: 10.1038/s41583-021-00466-4. 34045693

[bibr37-02698811241229524] Ramaekers JG Robbe HWJ O’Hanlon JF (2000) Marijuana, alcohol, and actual driving performance. Human Psychopharmacol 15: 551–558. DOI: 10.1002/1099-1077(200010)15:7<551::AID-HUP236>3.0.CO;2-P. 12404625

[bibr38-02698811241229524] Ramaekers JG . (2017) Drugs and driving research in medicinal drug development. Trends Pharmacol Sci 38(4): 319–321. DOI: 10.1016/j.tips.2017.01.006. 28283199

[bibr39-02698811241229524] Rekand T (2014) THC:CBD spray and ms spasticity symptoms: Data from latest studies. Europ Neurol 71: 4–9. DOI: 10.1159/000357742. 24457846

[bibr40-02698811241229524] Ronen A Gershon P Drobiner H , et al. (2008) Effects of THC on driving performance, physiological state, and subjective feelings relative to alcohol. Accid Anal Prev 40: 926–934. DOI: 10.1016/j.aap.2007.10.011. 18460360

[bibr41-02698811241229524] Ruheel MA Gomes Z Usman S , et al. (2021). Facilitators and barriers to the regulation of medical cannabis: A scoping review of the peer-reviewed literature. Harm Reduct J 18: 106. DOI: 10.1186/s12954-021-00547-8. 34649577 PMC8515704

[bibr42-02698811241229524] Schlag AK Hindocha C Zafar R , et al. (2021) Cannabis based medicines and cannabis dependence: A critical review of issues and evidence. J Psychopharmacol 35: 773–785. DOI: 10.1177/0269881120986393. 33593117 PMC8278552

[bibr43-02698811241229524] Sevigny EL (2021) Cannabis and driving ability. Curr Opin Psychol 38: 75–79. DOI: 10.1016/j.copsyc.2021.03.003. 33839427 PMC8106655

[bibr44-02698811241229524] Shiferaw BA Crewther DP Downey LA (2019) Gaze entropy measures detect alcohol-induced driver impairment. Drug Alcohol Depend 204: 107519. 31479863 10.1016/j.drugalcdep.2019.06.021

[bibr45-02698811241229524] Spindle TR Cone EJ Schlienz NJ , et al. (2019) Acute pharmacokinetic profile of smoked and vaporized cannabis in human blood and oral fluid. J Anal Toxicol 43: 233–258. DOI: 10.1093/jar/bky104. 30615181 PMC6676961

[bibr46-02698811241229524] Tement S Plohl N Horvat M , et al. (2020) Driving demands, stress reactivity, and driving behaviour: An interactional approach. Traffic Psychol Behav 69: 80–90. DOI: 10.1016/j.trf.2020.01.001.

[bibr47-02698811241229524] Turna J Balodis I Munn C , et al. (2020) Overlapping patterns of recreational and medical cannabis use in a large community sample of cannabis users. Compr Psychiatry 102: 152188. DOI: 10.1016/j.comppsych.2020.152188. 32653594

[bibr48-02698811241229524] Urtis I Borchart M Hasegawa M , et al. (2019) An update of current cannabis-based pharmaceuticals in pain medicine. Pain Ther 8: 41–51. DOI: 10.1007/s40122-019-0114-4. 30721403 PMC6514017

[bibr49-02698811241229524] Vandrey R Herrmann ES Mitchell JM , et al. (2017) Pharmacokinetic profile of oral cannabis in humans: Blood and oral fluid disposition and relation to pharmacodynamic outcomes. J Anal Toxicol 41: 83–99. DOI: 10.1093/hat/bkx012. 28158482 PMC5890870

[bibr50-02698811241229524] Veldhuijzen DS van Wijck AJM Wille F , et al. (2006) Effect of chronic nonmalignant pain on highway driving performance. Pain 122(1–2), 28–35. DOI: 10.1016/j.pain.2005.12.019. 16495013

[bibr51-02698811241229524] Verster JC Mets MA (2009) Psychoactive medication and traffic safety. Int J Environ Res Public Health 6: 1041–1054. DOI: 10.3390/ijerph6031041. 19440432 PMC2672393

[bibr52-02698811241229524] Verster JC Roth T (2011) Standard operation procedures for conducting the on-the-road driving test, and measurement of the standard deviation of lateral position (SDLP). Int J Gener Med 4: 359–371. DOI: 10.2147/IJGM.S19639. PMC3100218 21625472

[bibr53-02698811241229524] Wickens CM Watson TM Mann RE , et al. (2019) Exploring perceptions among people who drive after cannabis use: Collision risk, comparative optimism, and normative influence. Drug Alcohol Rev 38: 443–451. DOI: 10.1111/dar.12923. 30896069

